# Female social response to male sexual harassment in poeciliid fish: a comparison of six species

**DOI:** 10.3389/fpsyg.2015.01453

**Published:** 2015-09-29

**Authors:** Marco Dadda

**Affiliations:** ^1^Department of General Psychology, University of PadovaPadova, Italy; ^2^Centro di Neuroscienze Cognitive, Università di PadovaPadova, Italy

**Keywords:** mating behavior, mosquitofish, poeciliid fish, sexual harassment, sneak copulation

## Abstract

Sexual harassment is common among poeciliid fish. In some fishes, males show a high frequency of sneak copulation; such sexual activity is costly to the females in terms of foraging efficiency. In mosquitofish (*Gambusia holbrooki*), when males are present, the distance between females tends to decrease, and this behavior has been interpreted as an adaptive strategy to dilute the costs of male sexual activity. In this study, the tendency to reduce distance in the presence of a male has been investigated in females of six poeciliid species (*Girardinus metallicus, Girardinus falcatus, G. holbrooki, Poecilia reticulata, Xiphophorus hellerii*, and *Xiphophorus mayae*) that exhibit different male mating strategies and different levels of sexual activity. Results revealed large interspecific differences in the pattern of female aggregation. Females of species with a high frequency of sneak copulations tended to reduce their social distance in the presence of a male. By contrast, species that rely mainly on courtship showed little or no variation in social distance. The proportion of sneak copulations predicts the degree of variation in female social response, but the amount of total sexual activity does not, suggesting that the change in females' social distance when a male is present may indeed serve to reduce the costs of male sexual harassment.

## Introduction

Sexual conflicts arise when a mating strategy that maximizes the reproductive success of one sex is detrimental to the other sex (Parker, 1979). A typical sexual conflict concerns the number of mates, which usually has a stronger effect on males than on females (Bateman, 1948). Thus, male sexual harassment and coercive mating evolve as strategies to overcome the females' reluctance to mate (Clutton-Brock and Parker, 1995); females are expected to adopt counterstrategies in order to reduce the amount of unwanted mating, as has been described for several taxa (fish: Magurran and Seghers, 1994; insects: Stone, 1995; birds: Persaud and Galef, 2003; reptiles: Shine et al., 2004). Poeciliids, a group of freshwater fish with internal fertilization, represent a paradigmatic case of sexual conflict. Male poeciliids are probably the most ardent males among vertebrates; their sexual activity can reach one sexual act per minute (Bisazza et al., 1996; Houde, 1997). Females, however, can store sperm for months, and only a few copulations ensure the fertilization of all their eggs (Constantz, 1984). Such intense sexual activity by the males is costly to the females in terms of conspicuousness to predators, foraging efficiency, and offspring fitness (Pocklington and Dill, 1995; Pilastro et al., 2003; Dadda et al., 2005; Gasparini et al., 2012).

In the eastern mosquitofish, *Gambusia holbrooki*, it has been found that male sexual harassment can halve a female's foraging efficiency (Pilastro et al., 2003). The intensity of sexual harassment correlates negatively with male size because small males are more active. Usually, single females do not join males, but in the presence of a sexually active male, female mosquitofish change their social preferences and move either toward groups of males or toward males larger than the harassing one (Dadda et al., 2005; Agrillo et al., 2006). It has been proposed that all these female strategies have been favored by selection in order to minimize the costs of male sexual harassment (Dadda et al., 2008). Females can adopt strategies to reduce males' harassment by selecting habitats with fewer harassing males (Darden and Croft, 2008; Croft et al., 2009) or by joining shoals composed of other females (Cappozzo et al., 2008). Dadda et al. (2005) showed that the distance between two females decreases significantly when an active male is visible. More recently, it has been observed that, once harassed, females prefer to join larger groups and those composed of larger females (which are usually avoided)—probably because males prefer larger females males (Agrillo et al., 2008). Similarly, Brask et al. (2012) found that females actively decreased male harassment by associating with females more attractive than themselves. Finally, it has been suggested that female guppies can display plasticity in physiological traits to reduce the costs of sexual harassment. Female guppies exposed to high levels of male harassment showed increased swimming efficiency, spending less energy to move a given speed and distance (Killen et al., 2015).

However, factors other than sexual harassment might explain these variations in female aggregation. For example, it is possible that males, being more conspicuous, attract more predators, as shown in the guppy, *Poecilia reticulata* (Godin and McDonough, 2003), and in *Xiphophorus hellerii* (Hernandez-Jimenez and Rios-Cardenas, 2012); thus, females reduce swimming distance and join larger groups to avoid predation.

One way to test this hypothesis is to compare species that differ in frequency of sneak copulations. According to the sexual harassment hypothesis, variation in the social response should be more relevant in species with harassing males and should be less relevant in species with courting males. Poeciliids show considerable differences in mating behavior (Farr, 1989; Bisazza, 1993). Some species, such as mosquitofish, exhibit sneak copulations exclusively, without any form of courtship (Bisazza and Pilastro, 1997; Pilastro et al., 1997). Other species, such as guppies, exhibit both sneak copulations and courtship behavior, and the decision to rely on one of these tactics is often context-dependent (Houde, 1997; Ojanguren and Magurran, 2004; Gasparini et al., 2013; Locatello et al., 2015) and related to the male's phenotype (Magellan et al., 2005) or genital morphology (Evans et al., 2011; Gasparini et al., 2011). Finally, species like the green swordtail (*X. hellerii*) exhibit few or no sneak copulations and mainly rely on courtship (Ryan and Causey, 1989).

In this study, I compared six species of poeciliid fish: three species that rarely exhibit sneak copulation, mainly using courtship to mate, and three species that exclusively or predominantly use sneak copulation as a mating tactic. If the reduction in the swimming distance between females in the presence of a male represents a counterstrategy to sexual harassment, this strategy is expected to be more evident in species in which males harass females with a high frequency of sneak copulation.

## Materials and methods

### Study species and fish maintenance

**Figure 1:**
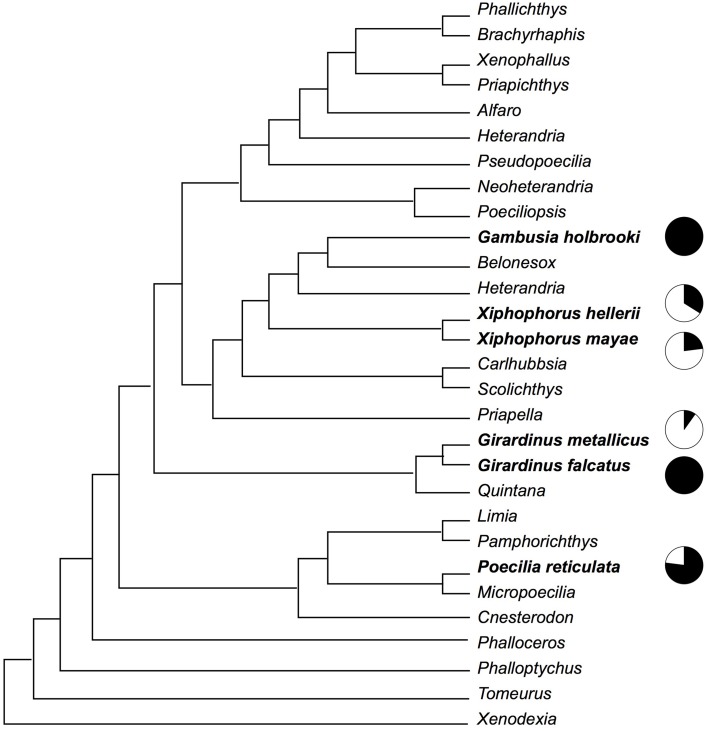
**Phylogenetic relationship among the six species observed in this study according to a recent phylogeny of the poeciliid group by Hrbek et al. (2007)**. Black in the pie charts indicates the proportion of gonopodial thrusts, while white indicates the proportion of courtship and nipping behavior.

This study involved six species of poeciliid fish: *G. holbrooki, Girardinus falcatus, Girardinus metallicus, P. reticulata, X. hellerii*, and *Xiphophorus mayae*. The phylogenetic positions of these species (see Figure 1) are approximately plotted on a recent phylogeny of the poeciliid group by Hrbek et al. (2007).

*G. holbrooki* were collected from Valle Averto, near the Venetian lagoon, in Italy, carried to the laboratory, and maintained in groups (20-25 individuals) in several stock tanks (100 L). *G. falcatus* and *P. reticulata* (Tacarigua River population) came from stocks maintained in our laboratory and were housed in groups of the same size as *G. holbrooki*. *G. metallicus* came from the Manfred Schartl Lab, University of Würzburg. A previous description of this species (Farr, 1980) revealed no courtship behavior in males. The population used in this study differs morphologically and behaviorally from the population that Farr described. Males present a black coloration that runs from the mouth along the underside of the fish into the gonopodium (an anal fin developed into a copulatory organ, Rosen and Bailey, 1963). These males tend to swim horizontally and close to the female's eye, showing their black bellies. This could be interpreted as a courtship behavior because it is quite hard for the male to produce sneaky copulations during this sequence (see also Kolluru et al., 2014).

*X. hellerii* and *X. mayae* originally came from a local pet shop and were obtained from Andrea Pilastro Lab, Department of Biology, University of Padova and maintained in larger tanks (150 L). All of the species observed in this study, except for *G. holbrooki*, were maintained in the lab for at least 10 generation. All tanks were provided with gravel, an air filter, and live plants (*Ceratophyllum spp*.) illuminated by two 15-W fluorescent lights and maintained at a constant temperature (25 ± 1°C) and photoperiod (0600–2000 h). Fish were fed twice a day with commercial food flakes and live *Artemia nauplii*. I used adult fish that were all sexually mature and had interacted with the opposite sex.

#### Animal ethics

The experiments comply with all laws of the country (Italy) in which they were performed (D.M. 116192), and the study was approved by the “Ministero della Salute” (permit number: 6726-2011). The methods were carried out in accordance with the approved guidelines.

### Variation in male sexual behavior

**Figure 2:**
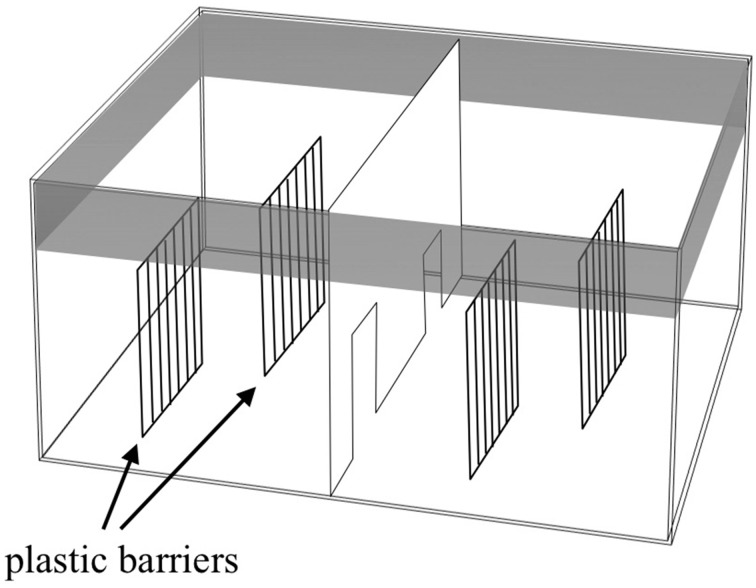
**Schematic representation of the apparatus used for evaluating the variation in male sexual behavior**.

The apparatus (Figure 2) was a large glass tank (65 × 65 × 40 cm) divided into two identical sectors by means of a transparent partition with two doors that allowed the subjects to move from one sector to the other. Two plastic barriers (25 × 30 cm) that simulated the plants in the natural environment were placed in the two sectors. Each barrier was composed of a series of elongated bars 1 cm wide and 0.5 cm apart. In this way, each sector was virtually divided into two identical sides. The apparatus was placed in a quiet, darkened room and surrounded with black curtains to prevent the fish from seeing the observer. For each observation, I measured the sexual behavior of three males with respect to three females. Compared to a single male–single female interaction, for this study, I decided to simulate a more ecological condition where females formed shoals of variable sizes and males interacted to get access to females.

In several species, males of different sizes differ in their reproductive tactics (Farr et al., 1986; Ryan and Causey, 1989). For this reason, for each observation, males were sorted according to their standard lengths as small, medium, and large (see Table 1). Also, females were sorted as small, medium, and large (see Table 1) even though during the data collection, I did not account for which female was engaged in sexual interaction. Twenty groups of six fish were observed (four groups of *X. mayae*, four of *P. reticulata*, four of *G. falcatus*, three of *G. metallicus*, three of *G. holbrooki*, and two of *X. hellerii*) for a total of 120 fish (60 females and 60 males). Subjects were inserted into the apparatus the day before the test and allowed to settle overnight.

**Table 1:** **Means ± standard deviation of the sexual behaviors recorded in the six species during 1 h of observation**.

**Species**	**SL females (mean /± SD)**			**SL males (mean /± SD)**			**Nipping**	**Gonopodial thrusts**	**Courtship**	**Total**	**Proportion of gonopodial thrust (%)**
	**Large**	**Medium**	**Small**	**Large**	**Medium**	**Small**					
*Xiphophorus mayae*	3.19 ± 0.5	2.5 ± 0.3	2.20 ± 0.2	2.91 ± 0.3	2.5 ± 0.2	2.17 ± 0.2	2.16 ± 4.04	1.33 ± 2.53	4.75 ± 5.29	8.25 ± 9.14	16
*Xiphophorus hellerii*	3.37 ± 0.3	2.75 ± 0.3	2.33 ± 0.2	3.13 ± 0.1	2.61 ± 0.1	2.15 ± 0.2	4.50 ± 3.98	4.83 ± 6.24	6.67 ± 8.18	16.00 ± 17.43	30
*Girardinus metallicus*	3.2 ± 0.1	2.76 ± 0.1	2.52 ± 0.2	2.22 ± 0.2	2.17 ± 0.2	2.0 ± 0.1	−	6.88 ± 6.48	58.33 ± 24.33	65.22 ± 26.81	11
*Girardinus falcatus*	3.27 ± 0.3	2.67 ± 0.2	2.35 ± 0.25	2.38 ± 0.25	2.34 ± 0.3	2.13 ± 0.2	−	12.50 ± 5.10	−	12.50 ± 5.10	100
*Poecilia reticulata*	2.49 ± 0.15	2.25 ± 0.2	1.84 ± 0.4	1.87 ± 0.2	1.73 ± 0.2	1.67 ± 0.2	−	37.75 ± 24.33	12.58 ± 17.40	50.33 ± 39.11	75
*Gambusia holbrooki*	2.83 ± 0.2	2.51 ± 0.2	2.18 ± 0.1	2.18 ± 0.2	2.06 ± 0.2	1.98 ± 0.2	−	55.55 ± 31.52	−	55.55 ± 31.52	100

Subjects were observed for two periods of 30 min each: a first period of 30 min during the first day and a second period of 30 min during the second day. Two cameras were used; one was placed approximately 1 m above the apparatus, and one was placed frontally at approximately 70 cm from the apparatus. From the video recordings, male sexual behavior was scored as follows: (1) body contact (nipping at the level of the female gonopore), (2) gonopodial thrusts (copulations and copulatory attempts), and (3) courtship behavior.

Nipping behavior can be described as a contact at the female genital pore and seems to precede copulation attempts in several species (Parzefall, 1969, 1973; Sumner et al., 1994). During the gonopodial thrusts, a male approaches the female from behind, turns the gonopodium 90°, and tries to insert it into the female's genital pore (Bisazza, 1993; Bisazza and Marin, 1995). Courtship behavior consists of a stereotyped swim, different from species to species, that allows the males to display their pigmentation or elaborate fins (Bisazza, 1993).

### Variation in female aggregation in the presence/absence of a male

The apparatus used for this test was the same as that described in previous experiments (Dadda et al., 2005, 2008). Briefly, the experimental tank was a circular arena (diameter, 65 cm) filled with 15 cm of water and illuminated with four 8-W fluorescent lamps. A hollow and transparent plexiglas cylinder (diameter, 19.5 cm; height, 16.5 cm) was placed in the center of the arena to enclose the stimulus. A second, opaque cylinder (diameter, 19 cm; height, 16.5 cm) was inserted into the first and suspended on a monofilament line attached to a pulley system allowing us to move it up and down. Two females of the same species matched in size (standard length difference ≤ 2 mm) were introduced into the arena and allowed to settle for 1 h. After this period, one active male was put into the central cylinder. The male could be hidden or shown to the females by movement of the opaque plastic cylinder up or down. A video camera, positioned about 2 m above the center of the apparatus, was used to record the trials, and video recordings were subsequently digitalized.

I tested eight female pairs of *G. falcatus*, eight of *G. metallicus*, eight of *P. reticulata*, seven pairs of *X. hellerii*, and seven pairs of *X. mayae*. I have included in the analyses the data of 12 pairs of *G. holbrooki* from a previous study (Dadda et al., 2005). The procedure was the same as that previously described (Dadda et al., 2005, 2008). Briefly, each trial consisted of eight observation periods, which were divided into four observations with the male visible and four with the male hidden. Each observation period lasted 30 min and was separated by a 10-min interval. Half of the trials started with a period where the male was visible in the central cylinder and half with the male kept hidden from the shoaling females. Three frames per minute were examined, and by means of a computer program (written in Delphi5 Borland), I measured the linear distance between the females. In total, 720 distance measurements for each trial (360 with the stimulus fish that were visible to the two experimental females and 360 with the hidden stimulus fish) were obtained. From these measures, I calculated the mean distance and the mean angle between the two experimental females for each 30-min period.

### Data analysis

Variation in male sexual behavior was analyzed using a linear model with fixed effects for the six species and for males' sizes (small, medium, and large), and the random effects model was used for each independently tested group.

Variation in female aggregation in the presence/absence of a male were analyzed by use of a repeated measures analysis of variance (ANOVA) in which the presence of the male and the succession of the observation periods were the within-group factors, while the order of presentation (whether the experiment started with the male that was visible or not visible to the females) and the six different species were the between-group factors.

The non-parametric Kendall's correlation was used to correlate intraspecific variation in female aggregation in response to the presence of the male, with the sexual behavior of males in the six species.

To express variation in social distance, I used a social distance index calculated as follows:

$$\begin{array}{l} (\text{mean distance with male not visible}\\ -\text{ mean distance with male visible})/\\ \text{mean distance with male not visible} \end{array}$$

A *post-hoc* test was performed using the LSD method. Statistics were done using SPSS 21 (IBM Inc., U.S.A.). Means are given ±SD (see Supplementary Material).

## Results

### Variation in male sexual behavior

Males of the six species significantly differed in the overall number of mating acts [*F*_(5, 14)_ = 11.834, *p* < 0.001]. Neither males' sizes nor the interaction between the two fixed factors was statistically significant [*F*_(2, 28)_ = 0.796, *p* = 0.461; *F*_(10, 28)_ = 0.905, *p* = 0.541, respectively]. Sexual activity ranges from a minimum of 8.25 sexual acts per male in *X. mayae* to 65.22 sexual acts per male in *G. metallicus* (see Table 1).

When the number of gonopodial thrusts was considered, the difference between males was significant [*F*_(5, 42)_ = 15.400, *p* < 0.001]. Neither males' sizes nor the interaction was significant [*F*_(2, 42)_ = 1.423, *p* = 0.252; *F*_(10, 42)_ = 0.744, *p* = 0.679, respectively]. Results were similar when the proportion of gonopodial thrusts over the total number of sexual acts was considered [*F*_(5, 14)_ = 34.941, *p* < 0.001]. Neither males' sizes nor interaction was significant [*F*_(2, 28)_ = 0.310, *p* = 0.736; *F*_(10, 28)_ = 1.598, *p* = 0.159, respectively].

### Variation in female aggregation in the presence/absence of a male

**Figure 3:**
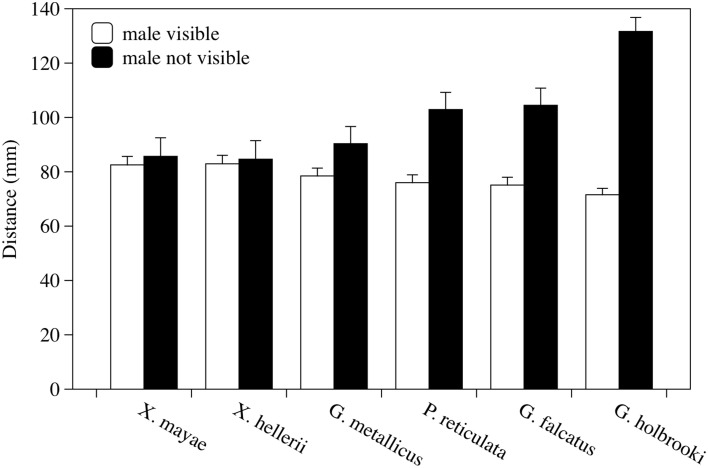
**Variations in distance between two experimental females according to whether a stimulus male was visible or not visible**. Mean distance in millimeters ± standard error is reported for each species observed. Each bar represents the mean of four observation periods.

The presence of the male significantly affected the female's behavior; females swam significantly closer together when the stimulus male was visible than when it was not visible [*F*_(1, 38)_ = 90.91, *p* < 0.001, Figure 3]. The interaction between the presence of the male and the different species was also statistically significant [*F*_(5, 38)_ = 17.91, *p* < 0.001]. A significant difference was found among females of six species [*F*_(5, 38)_ = 3.69, *p* = 0.008]. No other factors or interactions were significant. *Post-hoc* analyses showed that in *G. holbrooki*, the mean distance was significantly greater than it was in the other five species (LSD method, *p* < 0.050).

When the six species were examined in the same way separately, I found a significant decrease in social distance with a male present in *G. metallicus, G. falcatus, P. reticulata*, and *G. holbrooki* [*F*_(1, 6)_ = 14.217, *p* = 0.009; *F*_(1, 6)_ = 79.20, *p* < 0.001; *F*_(1, 6)_ = 34.90, *p* = 0.001; and *F*_(1, 10)_ = 57.45, *p* < 0.001, respectively] but not in *X. mayae* and *X. hellerii* [*F*_(1, 5)_ = 1.098, *p* = 0.343 and *F*_(1, 5)_= 4.060, *p* = 0.100, *p* < 0.001, respectively].

**Figure 4:**
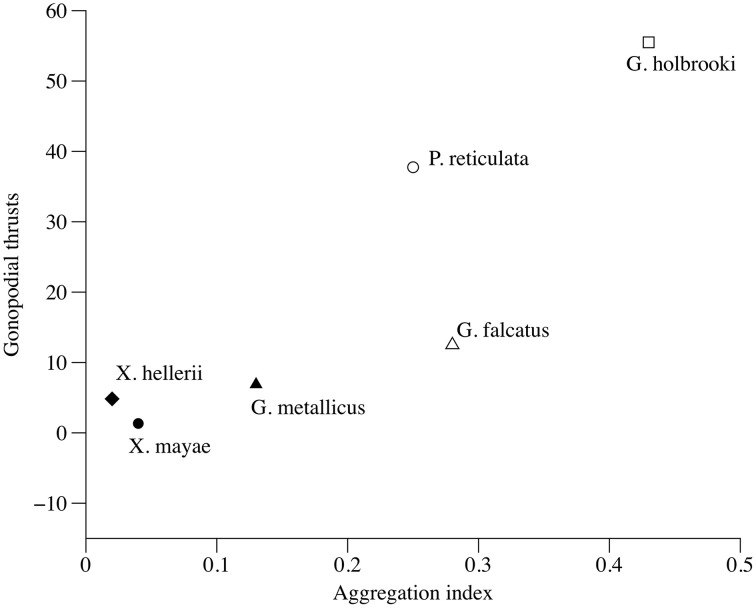
**Correlation between index of variation in female aggregation and total number of gonopodial thrusts**.

The total number of gonopodial thrusts correlates positively with female aggregation (τ = 0.733, *p* = 0.039, Figure 4), while the number of courtship acts and the total number of sexual acts were not significant predictors (τ = 0.276, *p* = 0.44 and τ = 0.333, *p* = 0.348, respectively) of a female aggregation pattern. The proportion of gonopodial thrusts on total sexual activity was also a significant predictor of aggregation in females (τ = 0.733, *p* = 0.039).

## Discussion

Several species of fish can modulate their gregarious behavior according to the costs and benefits of living in groups. A number of studies have shown that when a predator is present, individuals tend to prefer joining conspecific groups, as the dilution effect (among many other effects that render schooling beneficial, Pitcher and Parrish, 1993; Godin, 1997; Edenbrow et al., 2011; Killen et al., 2012) increases with group size. On the other hand, there are well-known costs, including increased competition for resources and mates as well as contagious disease (Reynolds and Gross, 1990). In *G. holbrooki*, females forage individually or in groups of variable sizes, whereas males move from one group to another (Bisazza et al., 2000). Males of this species are among the most ardent males in the animal kingdom (their sexual activity is so intense that they can complete one sexual act per minute, Bisazza et al., 1996), and this can greatly reduce female foraging efficiency (Dadda and Bisazza, 2006). It has been suggested that females prefer to join larger conspecific groups in order to dilute male disturbance (Agrillo et al., 2006). According to this, one might expect this tendency to be more pronounced in species in which males' sexual harassment is particularly intense compared to that of courting species.

The six species examined in this study showed significant differences in social distance. These different shoaling tendencies could be related to ecological differences. For instance mosquitofish, the species showing the largest inter-individual distance, are insectivorous surface feeders, and in the laboratory and field settings, they are often seen attacking neighboring females, probably in order to defend feeding territories. Another important factor that is likely to influence the shoaling tendency is the different predation risk to which one species is exposed in its habitat (Magurran and Pitcher, 1987).

Females of the six species showed a marked difference in social response when exposed to the sight of a conspecific male. Some species such as *X. mayae* and *X. hellerii* showed little or no variation in response to the presence of a male, while others such as *G. holbrooki* showed a large reduction in the distance at which they swam as soon as they were exposed to the male. However, the strongest difference between species is seen in inter-individual distances among female shoal members in species characterized by male coercive mating in the absence of a male. This result confirms that schooling has some costs, such as competition for food or parasite transmission (Pulliam and Caraco, 1984), and that females try to minimize these costs when males are absent. For example, in *G. holbrooki*, seeing another female instead of a male makes the females swim farther from one another (Dadda et al., 2005). Arguably, the counterbalance between costs and benefits may explain why females of these species tend to increase the distance at which they swim in unisexual schools and reduce it when harassing males are present. On the other hand, it is worth noting that males' presence in female social groups has been shown to disrupt female–female social networks (Darden and Watts, 2012).

Large interspecific differences can also be observed when sexual behaviors of the six species are considered. Sneak copulation in three species (*G. holbrooki, G. falcatus*, and *P. reticulata*) is consistent with previous findings (McPeek, 1992; Bisazza and Marin, 1995; Bisazza and Pilastro, 1997). On the contrary, in the remaining three species (*X. mayae, X. hellerii*, and *G. metallicus*), sneak copulations are extremely rare; males are brightly colored, and they rely almost exclusively on courtship behavior.

Why do females of certain species tend to join other companions when a male is visible while females of other species do not? Considering the relationship between males' mating tactics and females' social behavior, it is possible to note that the species in which males rely principally on courtship showed little or no variation in the swimming distance between the two females when a male was visible or not, whereas such variation in the swimming distance is particularly evident in the species that use forcible insemination as the main mating tactic. In fact, Darden and Watts (2012) showed that in *P. reticulata*, female social interactions are strongly affected by the presence of free-swimming harassing males and that females showed increased mobility and an alteration in space use when a male was present. More generally, sexual harassment appears to affect social associations; Darden et al. (2009) experimentally manipulated the degree of sexual harassment in female guppies and found that females exposed to sexual harassment had more disparate social networks and failed to developed social recognition.

The proportion of gonopodial thrusts during pre-copulatory behavior proved to be the best predictor of females' variation in swimming distance, while the total number of sexual acts was not a significant predictor. A second potential explanation for the reduced swimming distance observed between the two females when a male was visible is that males are often brightly colored, and their mating behavior is particularly intense, so they might be easily spotted by predators, thus resulting in an increased risk of predation for females (Pocklington and Dill, 1995; Darden and Croft, 2008). However, the results reported here did not support this hypothesis, and actually, in *X. mayae* and *X. hellerii*, females' swimming distance was not affected by the presence of a male.

Although the results of this study indicate an association between male mating strategies and female shoaling behavior, this conclusion may be limited by potential phylogenetic confounds. For example, two of the three less-coercive species belong to the same genus. A proper test of the hypothesis that females increase their aggregation in response to male harassment would require that phylogeny be taken into account (Harvey and Pagel, 1991), but due to the small number of species studied, the application of such methods to the data of this study would be premature. It is, however, interesting to note that two sister species, *G. falcatus* and *G. metallicus* (Figure 1), that show very different mating behaviors also show very different responses by females. Future research should expand the number of species investigated, extending the investigation to the other genera of the family *Poeciliidae* and using adequate methods to account for phylogenetic relationships.

One of the most relevant assumptions discussed above is that the increase in sexual harassment determines elevated costs for the female so that selection promotes strategies for reducing male sexual harassment, especially in species in which these costs are particularly relevant. This was tested in a comparative work by Plath et al. (2007) in nine species of poeciliid fish differing in their mating strategies. A series of differences exist between this work and that of Plath and colleagues that can potentially explain why in the present study sneak copulations represent a cost for females, whereas in the study of Plath and collaborators, it is all of the sexual activity that is costly for females. Here, a group of several females and males have been kept in the experimental tank for 24 h prior to the observation, whereas in the study of Plath and colleagues, a single female and a single male were observed 5 min after insertion in the experimental tank. It is possible that males of the present study were initially more active and exhibited more intense courtship behavior toward females they had never seen before and became less active after several hours. On the other hand, the frequency of sneak copulation appears to be comparable between the two studies as long as it is consistently high during the entire period of observation. An additional difference between this study and that of Plath and colleagues is that the latter used reduction in female feeding as the measure of costliness to females. As the authors have suggested, some of the reduction in feeding time in their experiment may be due to females' paying attention to courting males. The current study's measure of female response would not seem to have the same potential confound, which may contribute to the differences between these two studies.

Very recently, it has been showed that female guppies can display plasticity in physiological traits that reduce the costs of sexual harassment. Female guppies exposed to high levels of male harassment showed increased swimming efficiency, spending less energy on moving a given speed and distance (Killen et al., 2015). However, it is still difficult to clearly identify the potential benefits for females when mating with harassing males (Cordero and Eberhard, 2003; Tobler et al., 2011).

The present study provides evidence of large interspecific differences in the pattern of female aggregation in species that exhibit different male mating strategies. Results showed that same-sex schooling in females of species with a high frequency of sneak copulation is influenced by the direct benefits associated with a reduction in sexual harassment, whereas species that rely mainly on courtship showed little or no variation in social distance.

However, to date, the results reported here are confined to laboratory observation. Future studies should be conducted in the wild or at least in a semi-natural environment (see Köhler et al., 2011) in order to provide proper evidence on how males' behavior affects females' response. Finally, it is worth noting that sexual harassment also appears to be costly for males; in *Poecilia latipinna*, the intensity of sexual harassment influences the overall body fat content of males and females (Makowicz and Schlupp, 2013), an aspect that should be taken into account when evaluating the dynamics of female social response to male mating strategies.

### Conflict of interest statement

The author declares that the research was conducted in the absence of any commercial or financial relationships that could be construed as a potential conflict of interest.
